# Inherent flexibility of CLIC6 revealed by crystallographic and solution studies

**DOI:** 10.1038/s41598-018-25231-z

**Published:** 2018-05-02

**Authors:** Alisa Ferofontov, Roi Strulovich, Milit Marom, Moshe Giladi, Yoni Haitin

**Affiliations:** 1 Department of Physiology and Pharmacology, Sackler School of Medicine, Tel-Aviv University, Tel-Aviv, 6997801 Israel; 2 Sagol School of Neuroscience, Tel Aviv University, Tel Aviv, 6997801 Israel

## Abstract

Chloride intracellular channels (CLICs) are a family of unique proteins, that were suggested to adopt both soluble and membrane-associated forms. Moreover, following this unusual metamorphic change, CLICs were shown to incorporate into membranes and mediate ion conduction *in vitro*, suggesting multimerization upon membrane insertion. Here, we present a 1.8 Å resolution crystal structure of the CLIC domain of mouse CLIC6 (mCLIC6). The structure reveals a monomeric arrangement and shows a high degree of structural conservation with other CLICs. Small-angle X-ray scattering (SAXS) analysis of mCLIC6 demonstrated that the overall solution structure is similar to the crystallographic conformation. Strikingly, further analysis of the SAXS data using ensemble optimization method unveiled additional elongated conformations, elucidating high structural plasticity as an inherent property of the protein. Moreover, structure-guided perturbation of the inter-domain interface by mutagenesis resulted in a population shift towards elongated conformations of mCLIC6. Additionally, we demonstrate that oxidative conditions induce an increase in mCLIC6 hydrophobicity along with mild oligomerization, which was enhanced by the presence of membrane mimetics. Together, these results provide mechanistic insights into the metamorphic nature of mCLIC6.

## Introduction

The Chloride Intracellular Channel (CLIC) family forms a class of unusual and enigmatic intracellular proteins^1^. Consisting of six homologs in human (CLIC1-6), different CLIC members display a wide, tissue-specific differential distribution and have been shown as crucial participants in normal kidney function^2^, cell division^3^, bone resorption^4^ and hair cells stereocilia function^5^.

The overall globular architecture of the CLIC family is reminiscent to the omega-class of glutathione S-transferases (GST-Ωs)^6–8^. However, unlike GSTs, CLICs fail to demonstrate a non-covalent association with glutathione (GSH) in solution^6^. Moreover, CLICs were surprisingly shown to exhibit unmediated association with artificial membranes, resulting in formation of ion conducting pores^6,9–14^, as well as co-localize and interact with membrane proteins such as G protein coupled^15,16^, and ryanodine receptors^17^. These observations led to their classification as ‘metamorphic’ proteins^18^. Importantly, while the precise cellular trigger for this radical structural rearrangement remains elusive, the involvement of non-reducing and low pH conditions were suggested^11,13,14,19,20^. Nevertheless, direct structural evidence for such unique flexibility are largely missing.

Originally identified in rabbit gastric parietal cells and named parchorin^21^, CLIC6 is the most recent addition to the CLIC family^22^. With a full-length reading frame encoding 704 amino acid residues in humans, it is the longest isoform discovered. Additional to the canonical GST-Ω class carboxy-terminal CLIC domain, CLIC6 endows a unique amino-terminal, predicted to be highly flexible, which exhibits poor sequence similarity to any known protein. While its cellular function remains elusive, CLIC6 was shown to translocate from the cytosol to the apical membrane of gastric parietal cells in association with HCl secretion^23^ and to interact with dopamine D_2_-like receptors^15^. Hence, similarly to other CLICs, the functional role of CLIC6 likely requires radical conformational transitions, switching between globular-soluble and membrane-associated forms, under different environmental conditions.

Here, we set to explore the innate structural flexibility within the CLIC domain, using CLIC6 as a model. We report the crystal structure of the mouse CLIC6 canonical CLIC domain at 1.8 Å resolution, which reveals high homology to the canonical structures observed in other CLIC family members. Moreover, using small-angle X-ray scattering (SAXS) measurements we demonstrate that the crystal structure conformation of the CLIC domain of mouse CLIC6 is enriched in solution. However, ensemble optimization method (EOM) analysis of the SAXS data strikingly revealed the population of additional, extended conformations. Finally, we show that mCLIC6 undergoes a conformational change and oligomerization that depends on both oxidative conditions and the presence of lipids.

## Results

### CLIC6 adopts the canonical GST-Ω fold

CLIC6 consists of an atypically long N-terminus preceding a C-terminal CLIC module^22^. Sequence analysis revealed that this unique N-terminal segment likely forms a highly flexible polypeptide chain. Hence, in order to gain molecular insights into the metamorphic nature of the canonical CLIC domain of mouse CLIC6, we purified and analyzed its conserved CLIC module (mCLIC6; residues 363–597). mCLIC6 was highly soluble in solution and crystallized using standard techniques. The structure of mCLIC6 has been determined from two crystal forms at 1.8 Å (P 2_1_) and 1.9 Å (C 1 2 1) resolution, respectively (Table 1). Molecular replacement (MR), utilizing the human CLIC4 (PDB 2AHE)^14^ structure as a model, was used to solve both structures.

**Table 1:** Crystallographic statistics^a^.

**Data collection**		
Spacegroup	C121	P2_1_
Cell dimensions		
a,b,c (Å)	120.38, 51.64, 101.10	42.62, 68.99, 80.98
α,β,γ (°)	90, 119.62, 90	90, 95.14, 90
Beamline	ESRF ID23-1	
Wavelength (Å)	0.978	0.976
Resolution (Å)	1.8	1.7
Multiplicity	6.39	5.23
Completeness (%)	98.5	98.8
Mean I/σ(Ι)	11.6	12.8
R_merge_ (%)	7.8	6.5
**Refinement statistics**		
No. reflections (work/free)	32673/1720	35794/1884
Resolution range^a^	47.0–1.9	40.3–1.8
R_work_/R_free_	0.203/0.249	0.181/0.220
No. atoms		
Macromolecules	3370	3548
Ligands	33	15
Solvent	226	308
**average** ***B*** **-factor**		
Macromolecules	48.67	38.17
Ligands	108.43	133.51
Solvent	50.06	43.27
RMSD (bond lengths)	0.008	0.008
RMSD (bond angles)	0.90	0.91
Ramachandran outliers (%)	0	0

^a^After anisotropic scaling.

**Figure 1:**
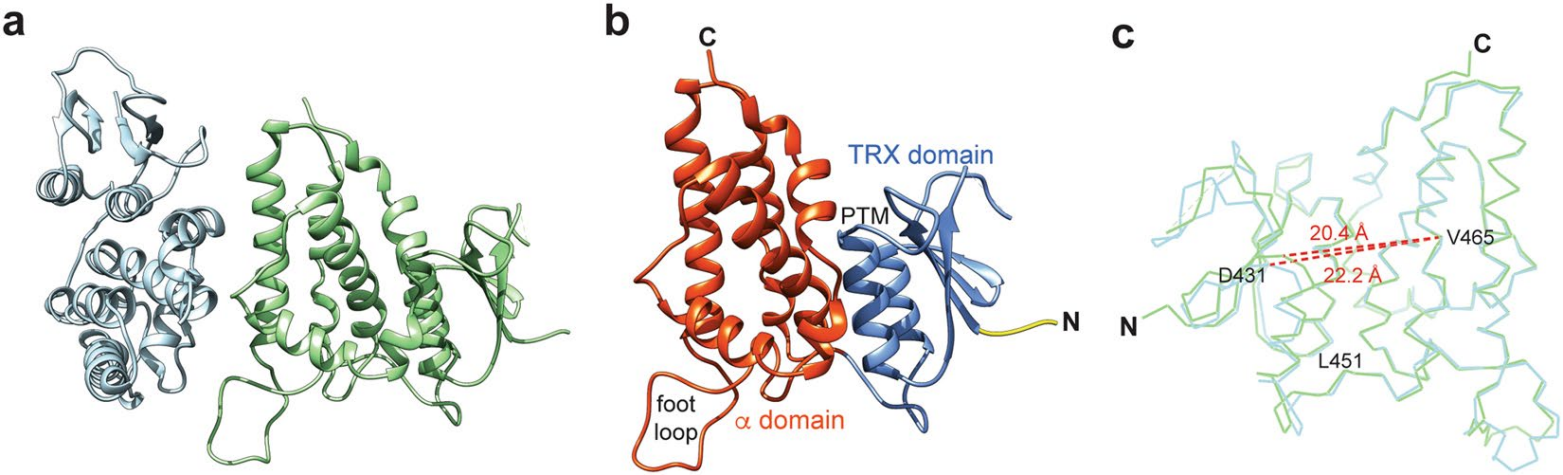
The structure of mCLIC6. (**a**) Cartoon representation of the asymmetric unit from the P 2_1_ crystal form showing chain A and B in cyan and green, respectively. (**b**) Cartoon representation of chain B. The structural motifs of the CLIC domain are labeled. (**c**) Superposition of the chains A and B from the P 2_1_ crystal form. Dashed red lines show distances between the indicated positions.

The overall structure, in both crystal forms, shows two nearly identical mCLIC6 subunits forming the asymmetric unit, each with 55 × 46 × 26 Å^3^ approximate dimensions (Fig. 1a, Supplementary Fig. S1, Table 1). Similar to other CLIC structures (Supplementary Fig. S2), mCLIC6 can be subdivided into a TRX (residues 363–442) and an α helical (residues 456–592) domains, connected by an intervening linker region (Fig. 1b). The TRX domain adopts a thioredoxin fold, consisting of four anti-parallel β strands (β1–β4), flanked by two α helices (α1 and α2). The α helical domain is formed by seven intertwined helices (α3–α9, Supplementary Fig. S3). Comparison of the relative chain conformations within the asymmetric unit yielded a root mean square deviation (r.m.s.d for all 226 C_α_ atoms) of 1.7 Å. However, overlaying the TRX or the α helical domains separately resulted in significantly superior alignments, with r.m.s.d of 0.6 Å and 0.5 Å, respectively, indicating an intrinsic inter-domain flexibility leading to structural plasticity. This property is unperturbed by the 13 amino acids linker segment (L443-H455), bridging the TRX and α helical domains. Moreover, using the superimposed α helical domain as a spatial anchor revealed a relative distance heterogeneity of 1.8 Å (D431-V465, C_α_) between the α helical and TRX domains of the two chains, accompanied by a 6.1° change in the inter-domain relative angle (D431-L451-V465, C_α_) (Fig. 1c).

**Figure 2:**
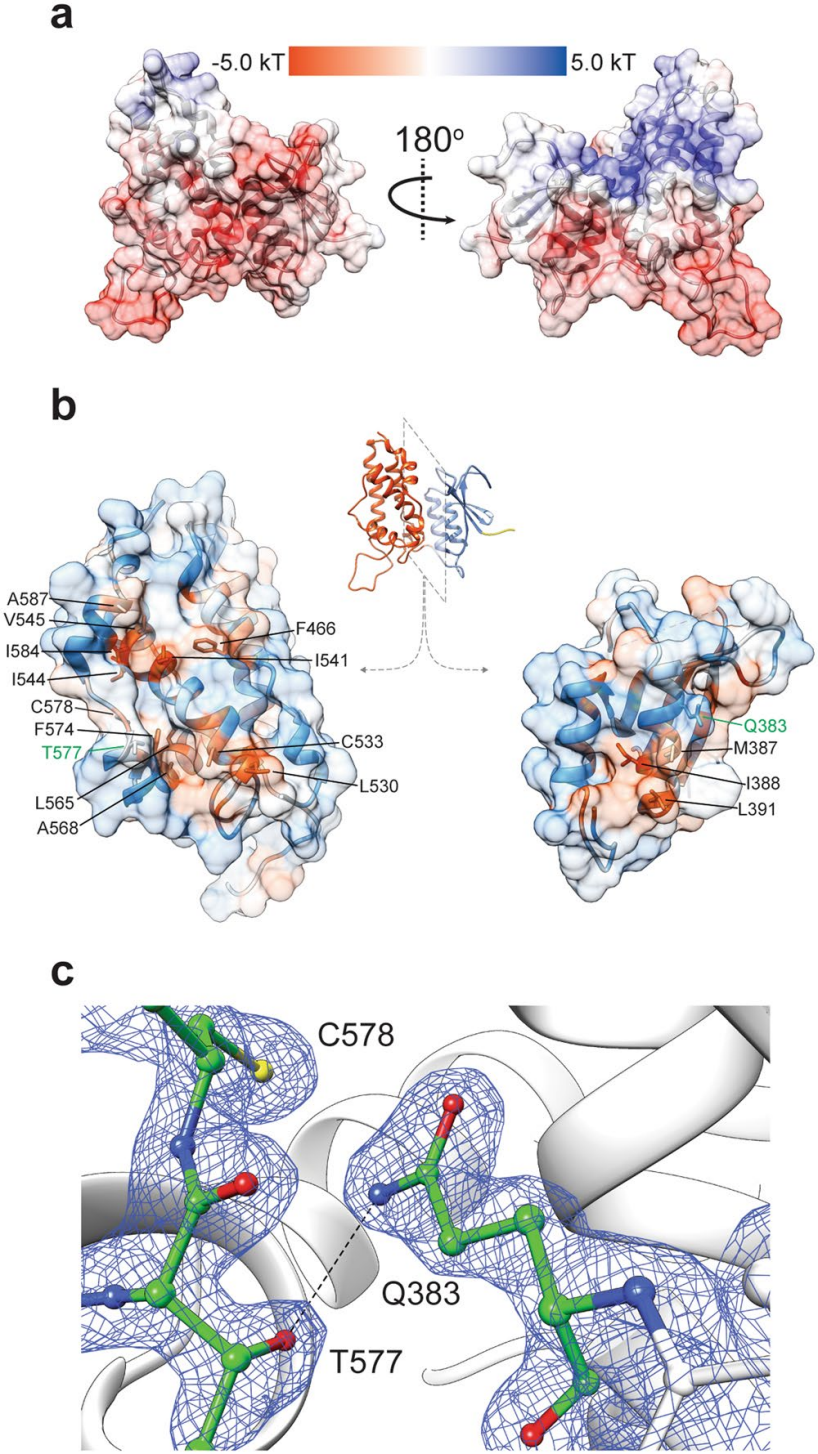
Surface and inter-domain interface analyses. (**a**) mCLIC6 electrostatic surface potential. (**b**) Inter-domain interface hydrophobicity analysis. Hydrophobic patches are shown in red. Positions participating in interface formation are indicated. (**c**) Detailed illustration of the Q383-T577 hydrogen bond (dashed black line), overlaid with 2*F*o – *F*c map contoured at 1*σ* (blue mesh). Indicated residues are shown as sticks and colored by element.

Next, we analyzed the surface potential of mCLIC6. Examination of the structure using Adaptive Poisson-Boltzmann Solver (APBS) showed that while most of the surface electrostatic profile of mCLIC6 is negatively charged, the inter-domain cleft, formed mostly by the amino terminal half of helix α1 from the TRX domain, and helices α4 and α6 from the α helical domain side, demonstrates a positive surface potential (Fig. 2a). Interestingly, the amino terminus of helix α1 contains the canonical ^379^Cys-Pro-Phe-Ser^382^ motif (Supplementary Fig. S3), crucial for ligand interaction in glutaredoxins^24^ and highly conserved in all CLICs. However, despite the structural homology with GST-Ω (r.m.s.d 3.2 Å, 195 C_α_ atoms), mCLIC6 does not exhibit detectable non-covalent interaction with glutathione in solution (not shown), similar to CLIC1^6^, suggesting that an alternative electrostatic mechanism may play a role in ligand specificity and association with possible native ligands.

**Figure 3:**
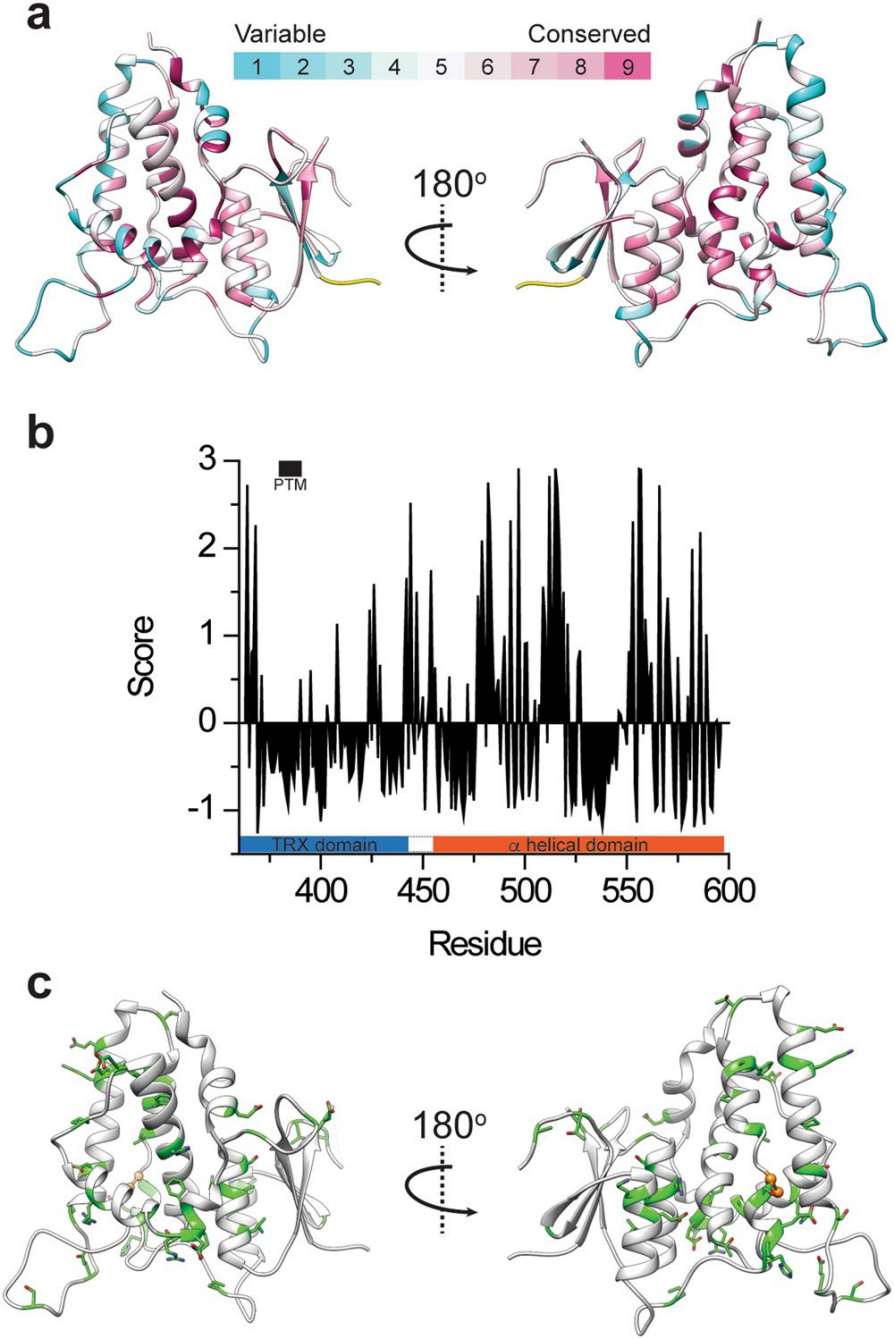
Conservation analysis and disease-associated mutation distribution in mCLIC6. (**a**) mCLIC6 structure colored according to the conservation score of each residue as determined by Consurf. (**b**) Conservation score for each residue. Negative values indicate conservation while positive values indicate variability. The sequence refers to the crystallized mCLIC6. (**c**) Cancer associated mutation positions (green sticks), mapped onto the structure of mCLIC6. A461, involved in both familial goiter and cancer, is shown as orange balls.

Additional to being covalently connected through a coiled linker, the TRX and α helical domains conceal an interface, consisting of ~1079 Å^2^ buried surface area composed from 23 and 29 residues, respectively. Surface hydrophobicity analysis using the Kyte-Doolittle scale revealed that two spatially segregated hydrophobic patches are involved in maintaining the structural stability of this inter-domain interface (Fig. 2b). Moreover, a single hydrogen bond involving side chain residues is formed between the highly conserved Q383 and T577, suggesting a possible unique structural significance for these positions in maintaining the globular conformation of the protein (Fig. 2c). Moreover, interacting residues have low relative B-factors and are highly, if not absolutely, conserved among CLIC isoforms and orthologs (Fig. 3a,b), a hallmark for their likely important structural or functional roles.

### Solution properties of mCLIC6

**Figure 4:**
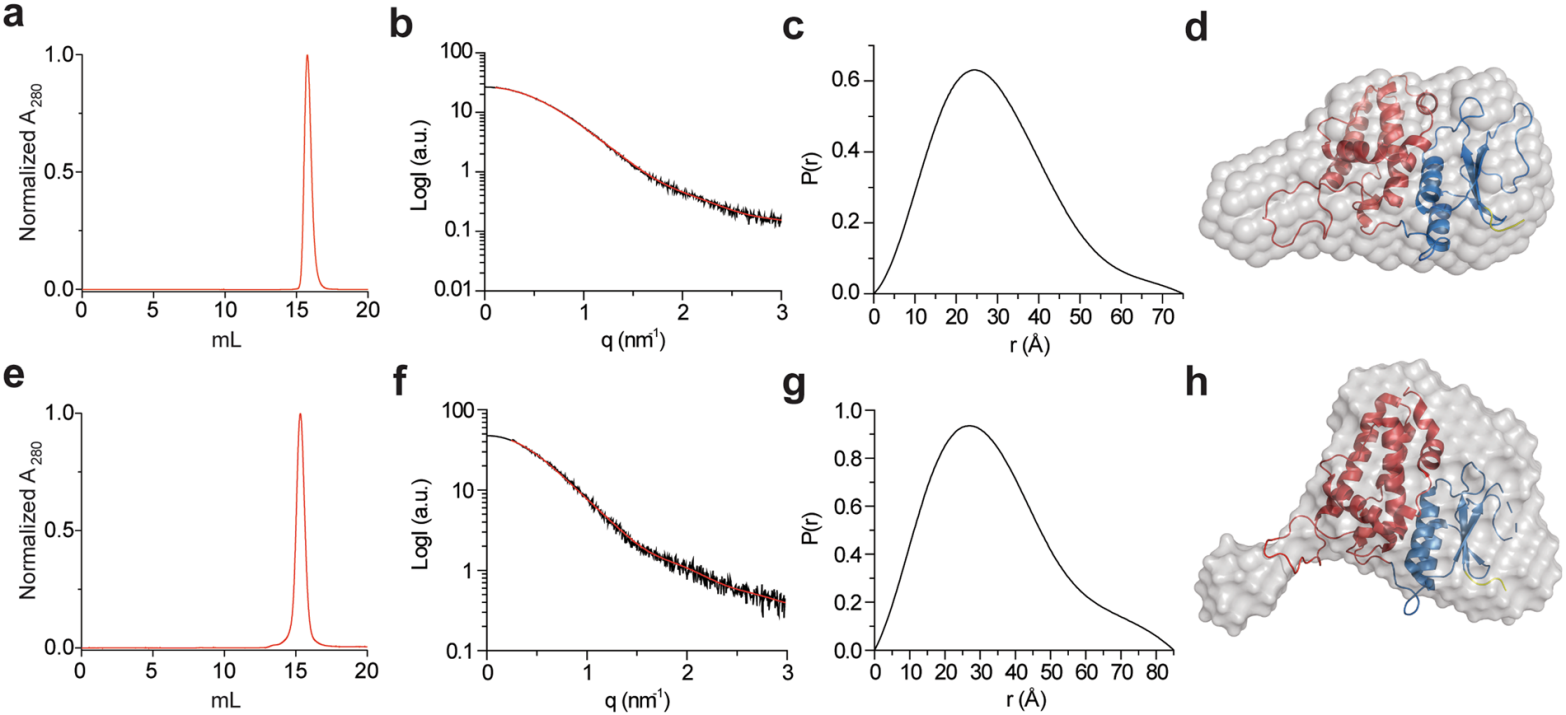
Solution properties of mCLIC6. (**a**) Size exclusion chromatography elution profile of mCLIC6-WT. (**b**) Experimental SAXS curves of mCLIC6-WT (black line) along with the fit obtained by GNOM (red line) used for *ab-initio* modelling. (**c**) Paired-distance distribution function of mCLIC6-WT determined using GNOM. (**d**) *Ab-initio* model of CLIC6-WT in solution (grey surface). (**e**) Size exclusion chromatography elution profile of mCLIC6-Q383A. (**f**) Experimental SAXS curves of mCLIC6-Q383A (black line) along with the fit obtained by GNOM (red line) used for *ab-initio* modelling. (**c**) Paired-distance distribution function of mCLIC6-Q383A determined using GNOM. (**d**) *Ab initio* model of CLIC6-Q383A in solution (grey surface). The structure of mCLIC6 was fit into the molecular envelopes using SUPCOMB. The TRX and α domains are presented as blue and red cartoons, respectively.

CLICs were suggested to belong to the metamorphic protein family, able to undergo radical conformational changes. However, to date, all the structural information available for CLICs samples a narrow range of the conformational space, focused at the globular-soluble conformation. Hence, in order to explore additional possible conformations that mCLIC6 may occupy, we proceeded with studying its structural properties in solution, an environment devoid of crystalline constrains, using analytical size exclusion chromatography (SEC) and SAXS analyses (Fig. 4). The gel filtration profile of mCLIC6 demonstrated a mono-dispersed elution profile, with a single peak reaching maximal absorbance at 15.8 ml (Fig. 4a), corresponding to an approximate M_r_ of ~29 kD, close to the calculated mass of a monomeric protein (27 kD). Hence, mCLIC6 consists of a homogeneous population in solution and does not form complex stoichiometric assemblies with high frequency under our experimental conditions.

Next, we utilized SAXS to detect possible relative reorientations of the TRX and α helical domains (Fig. 4b–d, Table 2). No aggregation-related issues nor radiation damage were observed throughout all SAXS experiments. GNOM was utilized to derive a paired distance distribution function (PDDF, Fig. 4c)^25^, followed by generation of an ab-initio model of the low-resolution molecular envelope in solution using DAMMIF^26^ (Fig. 4d). The molecular envelope revealed an elongated structure, which matches the crystal structure fairly well, but not perfectly (Fig. 4d).

**Table 2:** Data collection and scattering-derived parameters.

**Data collection parameters**		
	WT	Q383A
Beamline	ESRF BM29	
Beam geometry (mm^2^)	0.7 × 0.7	
Wavelength (Å)	1.0	
Q range (Å^−1^)	0.0025–0.5	
No. of frames	10	20
Exposure time per frame (seconds)^1^	0.5	1
Samples concentrations (mg/ml)	4.1, 1.6, 1.1	0.9, 0.43, 0.2
Temperature (°C)	5	
**Structural parameters**		
Rg (Å) [from P(r)]^1^	22.6 ± 0.004	26.3 ± 0.01
Rg (Å) (from Guinier)^1^	22.4 ± 0.1	25.8 ± 0.4
D_max_ (Å)^2^	75 ± 8	85 ± 9
Porod volume [from P(r)] (103 Å^3^)	42.53	50.75
NSD^3^	0.77 ± 0.04	0.55 ± 0.05
χ2 CRYSOL	3.8	5.2
χ2 EOM	1.0	1.1
**Software employed**		
Primary data reduction	AUTOMAR	
Data processing	PRIMUS	
Modelling	DAMMIF, EOM	

^1^±S.E.

^2^±10% (estimated range).

^3^NSD, normalized shape discrepancy for DAMMIF calculation.

**Figure 5:**
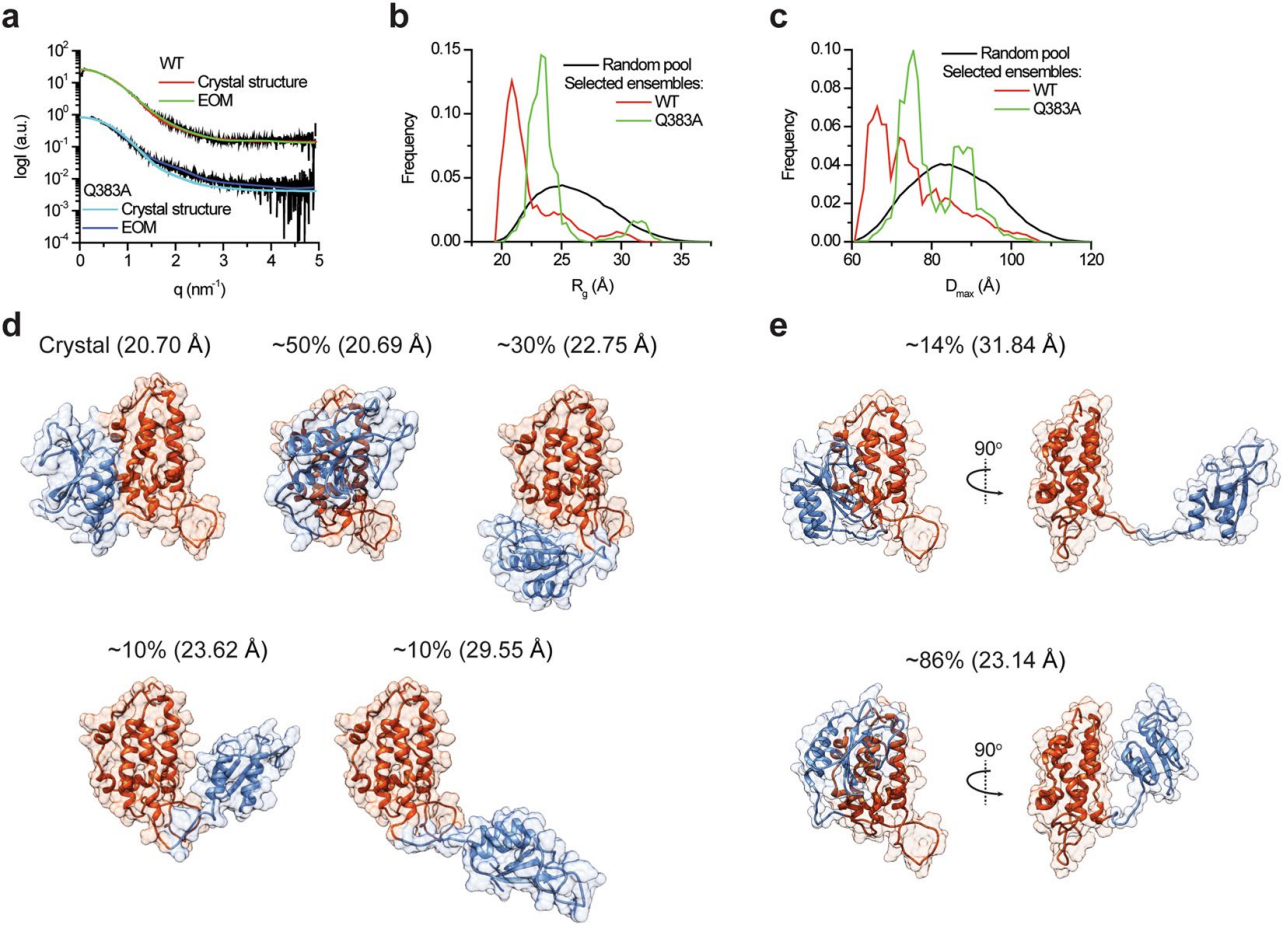
EOM analysis of mCLIC6. (**a**) Experimental SAXS curve of mCLIC6 (WT or Q383A; black lines) were fit using CRYSOL or EOM, as indicated. (**b**) Random Rg pool (black line) and EOM-selected ensemble distributions of mCLIC6-WT (red line) and mCLIC6-Q383A (green line). (**c**) Random D_max_ pool (black line) and EOM-selected ensemble distributions of mCLIC6-WT (red line) and mCLIC6-Q383A (green line). (**d,e**) Representative conformations of mCLIC6-WT (**d**) and mCLIC6-Q383A (**e**), found in the crystallographic and EOM analyses. The relative frequency of each conformation in the EOM analysis is indicated along the model Rg in parentheses. The TRX and α domains are presented as blue and red cartoons, respectively.

The imperfect match between the molecular envelope and the crystal structure motivated us to further assess the agreement between the crystallographic and solution structures. To that end, we used CRYSOL^27^ enabling the evaluation of the solution scattering originating from the crystal structure, and subsequently its fitting to the experimental SAXS curve. As shown in Fig. 5a, the crystallographic structure poorly matches the observed solution scattering curve, with χ^2^ = 3.8. Hence, we proceeded with assessing whether the observed scattering represents a mixture of conformations in solution. To explore this possibility, we employed an ensemble optimization method (EOM) analysis^28^ enabling determination of the conformational distribution. First, a random pool of conformations was obtained by allowing movement between the TRX and α domain based only on stereochemical restraints. Next, the ensemble of conformations that best fit the data was chosen using a genetic algorithm. The selected ensemble fits the data very well (χ^2^ = 1.0; Fig. 5a, Table 2) and the conformational distributions (Fig. 5b,c) show that the compact conformations, similar to those observed in the crystal structure, are predominant. However, additional more extended conformations also exist. Models of representative states, shown in Fig. 5d, reveal subpopulations (~20%) in which a significant perturbation of the interface between the domains occurs, exposing the hydrophobic patches as part of the surface area of the protein. Thus, in agreement with the structural flexibility observed in the crystal structure, the EOM analysis suggests that mCLIC6 samples multiple conformations in solution, a property that may hold significance for the metamorphic behavior of CLICs.

### Perturbation of the inter-domain interface enhances the flexibility of mCLIC6

As the EOM analysis of mCLIC6 indicated its propensity to occupy multiple conformational states in solution, we decided to examine whether the interface formed between the TRX and α domains plays a role in stabilizing the crystal structure conformation. While this interface demonstrates highly hydrophobic surface properties (Fig. 2b), close inspection of its structure revealed a single inter-domain hydrogen bond, bridging the putative transmembrane region (PTM; helix α1), localized to the TRX domain (Q383), and the C-terminal end of helix α8 of the α domain (T577) (Fig. 2c). Hence, in order to determine the significance of this interaction for the integrity of the globular form of mCLIC6, we introduced a hydrogen bond perturbing mutation (Q383A) and employed identical SAXS analyses. Unlike the wild-type (WT) mCLIC6, Q383A demonstrated reduced expression and solubility, and was crystallization resistant. The gel filtration profile of Q383A demonstrated a mono-dispersed elution profile, similar to the WT protein, although reaching maximal absorbance after ~15.2 ml, suggesting altered column migration properties (Fig. 4e). Interestingly, GNOM-derived PDDF analysis of Q383A resulted in increased D_max_ (Figs 4f and g, Table 2). Moreover, the molecular envelope calculated for Q383A was in poor agreement with the crystal structure (Fig. 4h), further confirmed using CRYSOL (χ^2^ = 5.2; Fig. 5a, Table 2). Importantly, EOM analysis of Q383A revealed sampling of a different conformational space region (Fig. 5b and c), with predominant and more discrete elongated conformations frequently populated (Fig. 5e). Hence, perturbation of the hydrogen bond between the TRX and α domains results in significant changes to the structure of mCLIC6.

### Exposure to oxidative conditions results in hydrophobicity increase of mCLIC6

**Figure 6:**
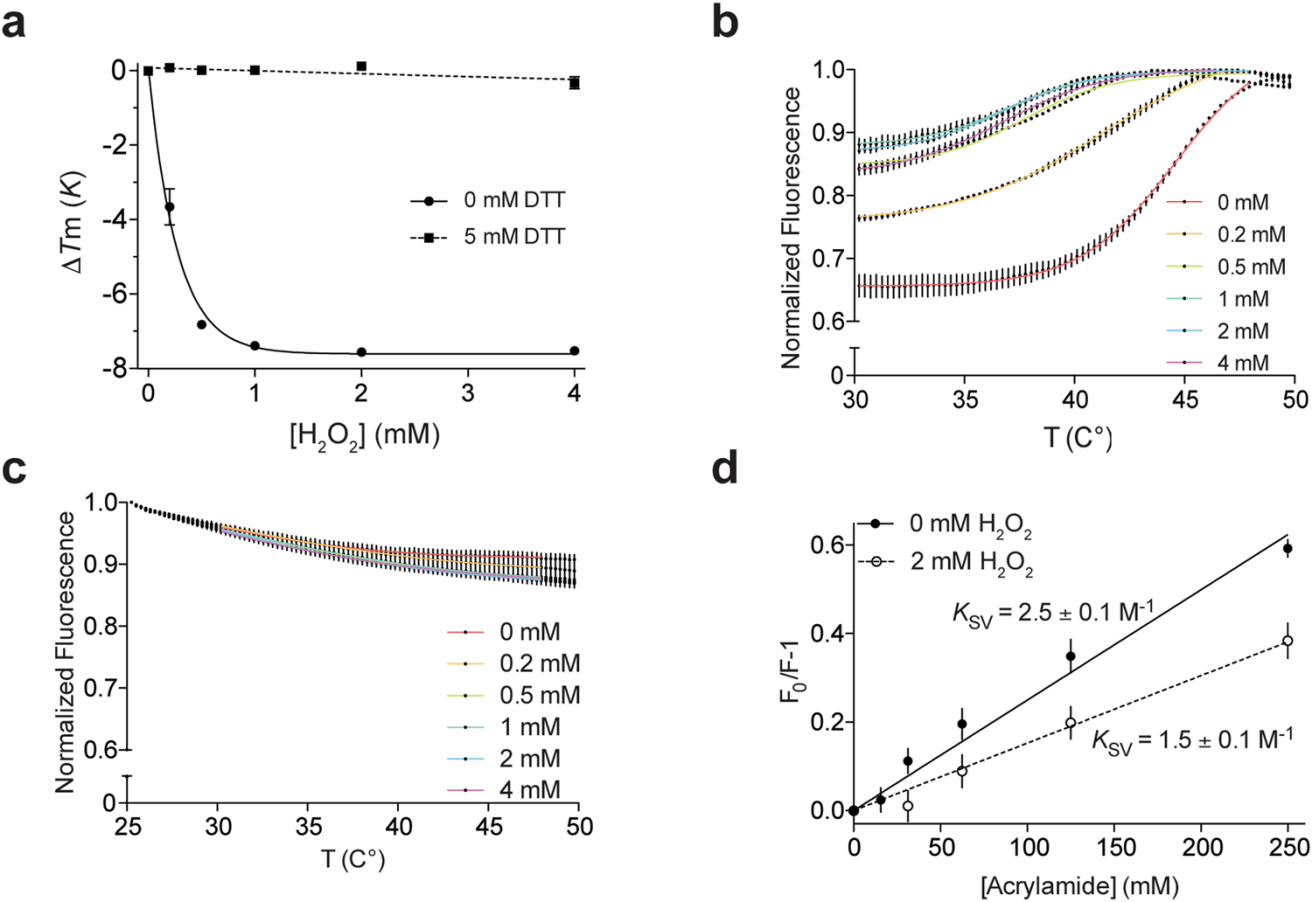
mCLIC6 response to oxidative environment. (**a**) The effect of H_2_O_2_ on mCLIC6, measured using differential scanning fluorimetry in the presence or absence of 5 mM DTT, and presented as *T*m difference. (**b**) Normalized fluorescence-temperature relation of mCLIC6 in the presence of increasing concentrations of H_2_O_2_. Boltzmann fits used to calculate *T*m are colored according to H_2_O_2_ concentrations. (**c**) Normalized fluorescence-temperature relation of mCLIC6-Q383A in the presence of increasing concentrations of H_2_O_2_. (**d**) Stern–Volmer plots for mCLIC6 tryptophan fluorescence quenching by acrylamide in both the absence and presence of H_2_O_2_.

While mCLIC6 mostly adopts a cytosolic globular conformation in solution (Fig. 4), we wondered whether environmental cues can facilitate structural rearrangements. Hence, we proceeded with examining the effects of oxidative conditions on mCLIC6 behavior in solution. To this end, we employed differential scanning fluorimetry, using the SYPRO Orange thermal shift assay (TSA)^29^, to quantify the effect of H_2_O_2_ on the denaturation temperature of mCLIC6, under native or reducing conditions (Fig. 6a). In the absence of H_2_O_2_, mCLIC6 exhibited moderate 44.2 ± 0.05 °C *T*m, and a 2 ± 0.06 slope, indicating the protein is stable in our experimental conditions and undergoes a sharp denaturation transition between folded and unfolded state. However, exposing the protein to increasing concentrations of H_2_O_2_ demonstrated an unexpectedly high sensitivity, with 50% of the total *T*m decrease already achieved at 0.19 ± 0.06 mM (Fig. 6a). Importantly, addition of 5 mM DTT completely abolished the oxidation-dependent *T*m shift (Fig. 6a, Supplementary Fig. S4), pointing towards the involvement of cysteine oxidation in perturbing the stability of mCLIC6.

Additional to the oxidation-mediated *T*m shift of mCLIC6, the TSA analysis revealed an H_2_O_2_-dependent increase in the basal fluorescence signal (Fig. 6b), decreasing the overall amplitude of the fluorescence change. As high initial fluorescence signal in this assay may suggest the presence of a partially unfolded state, or an exposure of hydrophobic patches in the native state, which might interact with the dye^30^, we proceeded by examining whether this effect results from perturbations to the secondary structure of the protein. Far-UV circular dichroism spectroscopy (CD) revealed that, similar to CLIC1^31^, mCLIC6 demonstrates an α helical-enriched fold in solution (34.35 ± 1.37%, n = 2), consistent with its crystal structure (Fig. 1). Incubation of the protein with 2 mM H_2_O_2_ did not result in significant spectral changes, with the α helical proportion unperturbed (31.7 ± 2.55%, n = 4, *p* > 0.05), indicating that the secondary structure of the protein is preserved under oxidative conditions (Supplementary Fig. S5). TSA analysis of Q383A mutant, that was shown to occupy more elongated conformations (Figs 4 and 5), demonstrated maximal fluorescence already at 25 °C, which was insensitive to H_2_O_2_ (Fig. 6c). Finally, we also probed oxidation-dependent conformational rearrangements using acrylamide-mediated tryptophan quenching assay (Fig. 6d). Acrylamide is a water soluble quencher of tryptophan fluorescence, used for probing the surface accessibility of tryptophan residues^32^. mCLIC6 contains two endogenous tryptophan residues, W390 and W562, which localize to the TRX and α domains, respectively. Similar to CLIC1^32,33^, Stern–Volmer plot analysis demonstrate that while exposure of mCLIC6 to increasing concentrations of acrylamide resulted in a progressive quenching of tryptophan fluorescence (K_SV_ = 2.5 ± 0.1 M^−1^), addition of 2 mM H_2_O_2_ reduced the acrylamide-induced tryptophan quenching efficiency (K_SV_ = 1.5 ± 0.1 M^−1^), indicating reduced tryptophan accessibility under oxidative conditions. Hence, these observations suggest that mCLIC6 undergoes a conformational rearrangement, triggered by oxidative environment, which possibly leads to exposure of nascent hydrophobic domains.

### mCLIC6 undergoes oligomerization in the presence of lipids and oxidative conditions

**Figure 7:**
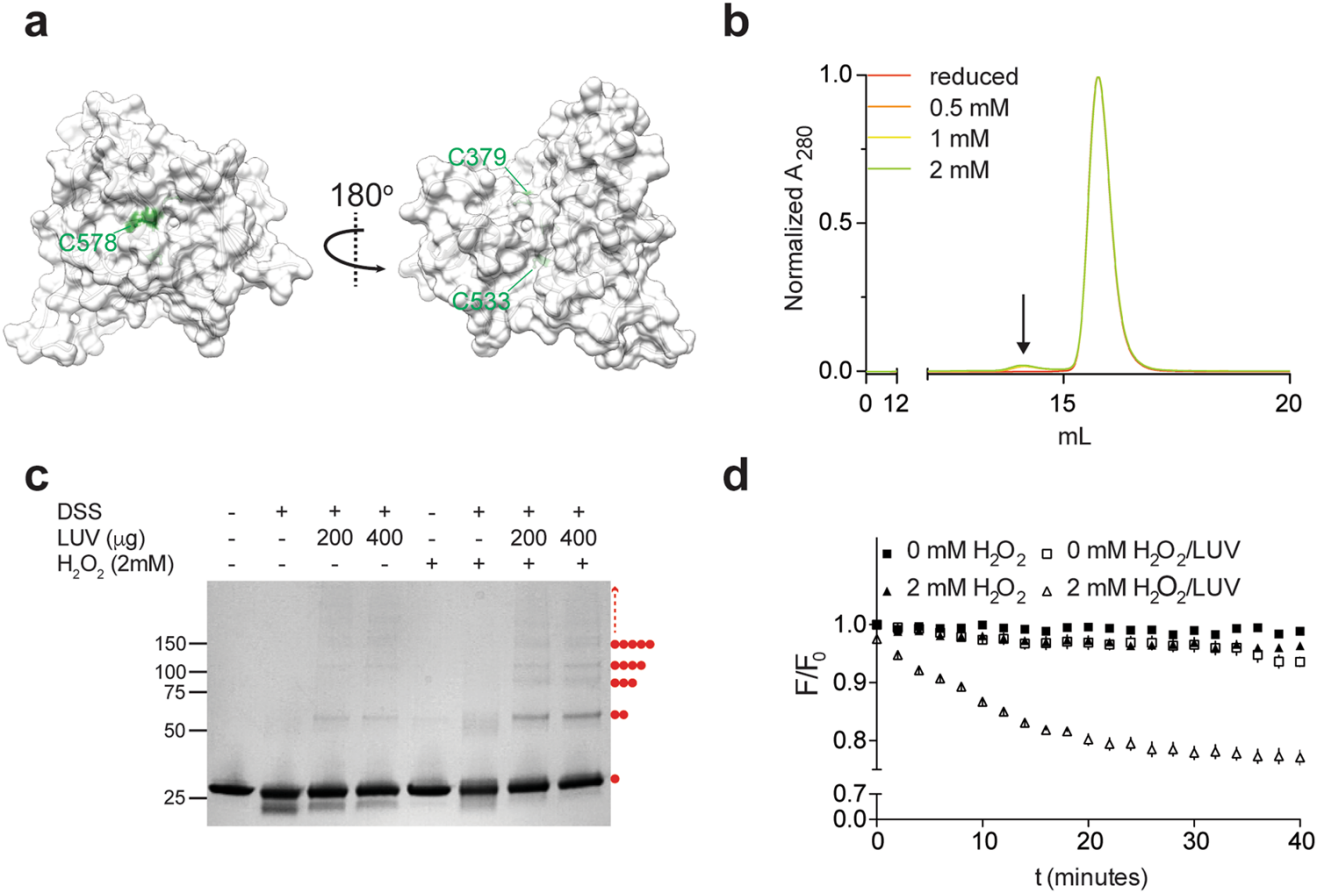
mCLIC6 oligomerization and membrane association. (**a**) Cysteine exposure (green), demonstrated using surface representation of mCLIC6 structure (grey). Note the C533 is inaccessible to the external solution. (**b**) Analytical size exclusion chromatography in the presence of increasing H_2_O_2_ concentrations. (**c**) SDS-PAGE of mCLIC6, following DSS-mediated cross-linking in the presence of H_2_O_2_ and LUVs. Red circles represent visible oligomeric states of the protein (n = 3). (**d**) mCLIC6 tryptophan fluorescence decay kinetics in the absence or presence of H_2_O_2_ and LUVs.

CLICs metamorphosis from globular to transmembrane conformations was suggested to involve the translocation of the β1-α1-β2 motif in the N-terminal region of the TRX domain into the membrane plane, resulting in a transmembrane spanning segment^2,6,9,20,34–36^. However, in order to form membrane-immersed traversing conductive pores, CLICs must also undergo an obligatory multimerization process. In CLIC1, changes in redox conditions or pH facilitate conformational changes within the TRX domain, resulting in the formation of a stabilized, yet soluble, dimeric arrangement^20^. Importantly, this dimerization was shown to be mediated by mere hydrophobic interactions, rather than a covalent association. Nevertheless, of the three endogenous cysteines present in mCLIC6, C379 and C578 show significant solvent accessible surface area (Fig. 7a), poising them for participation in oxidation-mediated conformational rearrangements. Therefore, we asked whether cysteine oxidation is sufficient to induce oligomerization. Purified mCLIC6 was exposed to increasing concentrations of hydrogen peroxide (H_2_O_2_), ranging from 0 to 2 mM, for 10 minutes incubation periods. Following these treatments, samples were sequentially loaded onto an analytical gel filtration column, equilibrated in running buffer absent of reducing or additional oxidative agents (see material and methods). Regardless of the H_2_O_2_ concentration, the majority of mCLIC6 displayed a monomeric elution profile, with a small sub-population eluting at a volume corresponding to a dimeric arrangement. Thus, despite the oxidation-dependent structural modification, these conditions alone are insufficient to induce major oligomerization (Fig. 7b), indicating that intermolecular disulfide bridges are not formed with high frequency under mere exposure to oxidative conditions.

Next, we asked whether the presence of membrane mimetics can modify the solution stoichiometry of mCLIC6 and facilitate its oligomerization. In order to achieve this goal, an approach that enables spatial immobilization of given stoichiometric states was required. Therefore, we resorted to a cross-linking strategy, performed in the presence or absence of large unilamellar vesicles (LUV), under different oxidative conditions, to resolve possible high molecular weight assemblies by SDS-PAGE (Fig. 7c). Incubation of mCLIC6 with 1 mM of disuccinimidyl suberate (DSS), a bifunctional amine-reactive N-hydroxysuccinimide (NHS) ester cross-linker, containing an eight-carbon spacer arm, resulted in the appearance of an additional highly mobile protein band, likely representing a partially auto-cross-linked species, and did not expose any high molecular weight oligomeric states, indicating a monomeric distribution in solution, similar to the SAXS analysis (Fig. 4). Surprisingly, co-incubation of mCLIC6 with DSS, together with increasing concentrations of LUVs, resulted in a moderate oligomerization, most evident by the emergence of weak yet visible protein bands, corresponding to higher molecular weight species. Moreover, inclusion of 2 mM H_2_O_2_ in the reaction mixture facilitated the formation of pronounced and discrete high-order oligomers, which highlight the involvement of both oxidative conditions and the presence of lipid in this process (Fig. 7c).

In order to further elucidate the interaction of mCLIC6 with the membrane, we performed time-course measurements of tryptophan fluorescence in the presence or absence of H_2_O_2_ and LUVs. Tryptophan fluorescence is strongly influenced by its (or the protein’s) local environment in terms of wavelength and intensity^37^. Hence, it is widely used as a tool to monitor changes in protein structure, environment and dynamics. Consistent with mCLIC6 cross-linking (Fig. 7c), incubation of the protein with H_2_O_2_ or LUVs separately resulted in marginal fluorescence decrease (3.1 ± 0.4% and 3.5 ± 1.2%, respectively; t = 30 minutes). However, co-incubation with H_2_O_2_ and LUVs led to a robust decay of the tryptophan fluorescence (21.5 ± 1.1%; t = 30 minutes), suggesting that both oxidation and the presence of membranes facilitate significant changes to the local environment of the two tryptophan residues of mCLIC6 (Fig. 7d). Hence, mCLIC6 is capable of undergoing oligomerization, which entails interaction with membranes, and is enhanced by oxidative environment.

## Discussion

CLIC proteins are a unique family of metamorphic proteins able to adopt both a globular soluble form as well as an integral membrane conformation. This unusual ability was attributed to a large conformational change within the canonical CLIC domain. In this paper, using a combination of structural, biophysical and biochemical methods we unveil great flexibility within the CLIC domain of CLIC6. Additionally, we show that oxidative conditions likely increase its hydrophobicity, and that a combination of oxidation and lipids facilitates oligomerization.

Over the past two decades, several crystal structures of CLICs have been resolved^6,14,20,38–41^. Nevertheless, despite the wealth of structural information, the metamorphic abilities of these proteins remained largely unattended. Hence, in order to expand our understanding of this widely expressed, yet functionally elusive, protein family, we sought to determine the crystal structure of mCLIC6. As a stable and well-behaved purified protein (Fig. 4a), mCLIC6 readily crystallized, in two discrete space groups, which diffracted up to 1.8 Å (Table 1). Dali server^42^ superimposition analysis of selected pruned atom pairs from the mCLIC6 structure and all the available CLIC structures found in the protein data bank revealed a close conformational correlation, with root mean square deviation (RMSD) ranging from 1.3 Å (CLIC4; PDB 2AHE and 2D2Z) to 2.2 Å (EXC-4; PDB 2YV9). While the precise cellular roles of CLICs await determination, their common ‘CLIC-fold’ must serve a yet to be resolved purpose. Indeed, rarely occurring mutations in CLIC2^43^, CLIC5^44^ and CLIC6^45^, have been associated with heart disease, deafness and familial goiter, respectively, further highlighting the functional importance of this family.

Interestingly, CLIC6 expression profile was shown to be altered in breast cancer^46^. Indeed, several members of the CLIC family were shown to overexpress in several tumor types^47,48^. Rummaging through the Catalogue of Somatic Mutations in Cancer (COSMIC)^49^ reveals 93 cancer-associated mutation annotations in the longest splice variant of CLIC6, with 58% classified as substitution missense mutations in positions demonstrating mostly high degree of sequence conservation (Supplementary Fig. S6). Mapping these positions onto the mCLIC6 structure (Fig. 3c) revealed that while cancer-associated mutations are scattered throughout the protein sequence, most of helix α3 (residues 456–461) and the amino terminal half of helix α8 (residues 571–574) show a significant mutational cluster, highlighting a possible functional role of these regions. Moreover, A551T in the human CLIC6, a position corresponding to A461 in mCLIC6, was found to be associated with familial goiter^45^, and also detected in a colon adenocarcinoma patient^49^. Situated immediately C-terminal to helix α3, this substitution may point towards a pathophysiological mechanism involving destabilization of the α helical domain. Finally, while none of the cancer associated positions appear to directly mediate the formation of the inter-domain interface, several localize to the PTM^35^ (R384, L385 and L389) and β1-α1 loop (G373 and G377), and helix α6 (H540) of the TRX and α helical domains, respectively. G467E, found in two malignant melanoma cases^49^ (G377 in mCLIC6) resides in close proximity to E673 (E583 in mCLIC6), indicating a possible structural pathology involving steric-hindrance (C_α_-C_α_ 5.9 Å).

Surface electrostatics analysis of mCLIC6 revealed an asymmetric charge distribution, with extensive positively and negatively charged pockets found in the cleft between the TRX and α domains and next to the foot loop, respectively (Fig. 2a), similar to other family members. Importantly, the mapping of cancer-associated positions onto the structure of mCLIC6 reveals an apparent mutational cluster localized within this inter-domain cleft. One of these positions, R384, is highly conserved and crucial for maintaining the thiol reactivity of the Cys-Pro-Phe-Ser motif ^6^. Moreover, it was found to be mutated in cases of colon adenocarcinoma (human CLIC6 R492H)^50^. Intriguingly, the structure reveals that R384 forms a salt bridge with E436 (2.87 Å_NH2-OE2_), an additional cancer-associated position found in malignant melanoma^49^, raising the possibility that disease-related perturbation of this interaction may contribute to an unresolved pathophysiological mechanism. Thus, this inter-domain cleft may act as receptor for yet to be determined factors, which in turn contribute to vital cellular roles.

Numerous observations justify the classification of CLICs as metamorphic proteins. Indeed, the strongest support for this unusual characteristic arises from their ability to elicit electrical conduction in reconstituted systems^6,9–14^ and give rise to IAA-94-sensitive Cl^−^ efflux^13,51,52^, a property which likely involves extensive conformational changes. Additionally, CLICs were shown to participate in various membrane-related processes such as actin-dependent membrane remodeling^11,21,23,53^, as well as vacuole formation and fusion^2^. Hence, the question remains – how do CLICs, as cytosolic globular proteins, manage to associate with membranes and facilitate the generation of quantifiable ionic currents *in vitro*? Using SAXS we exposed the flexible nature of mCLIC6 in solution, providing possible mechanistic clues for this process (Figs 4 and 5). Our EOM analysis demonstrates that mCLIC6 occupies multiple segregated forms in solution (Fig. 5), some of which may serve as ‘conformational precursors’ for subsequent targeted interactions. We suggest that the weakly conserved inter-domain linker (residues 443–455; 0.34 average normalized Consurf score^54^; Fig. 3a and b) may serve as a hinge between the TRX- and α-domains, possibly playing a key role in CLICs’ conformational plasticity. Furthermore, perturbation of the single hydrogen bond between Q383 and T577 (Fig. 2c), found within the TRX and α domains domain interface, resulted in a significant population shift, manifested as an emergence of predominant and distinct elongated species (Figs 4 and 5). In agreement, while properly folded, the introduction of Q383A radically altered the TSA profile of mCLIC6 rendering it highly accessible for interaction with SYPRO Orange even at 25 °C (Fig. 6c). Thus, we hypothesize that this ‘natural flexibility’, augmented by the inter-domain interface perturbation, together with oxidation-induced structural changes, prime CLICs for subsequent membrane interaction. In support, the PTM of CLIC1 and CLIC4 was shown to translocate from the cytosol to the membrane environment in response to oxidative^12,20^ or acidic^31^ conditions.

Nevertheless, while CLICs’ high innate flexibility is likely a necessary prerequisite for interaction with the membrane, the presence of only a single predicted PTM per molecule renders CLIC subunits insufficient to generate transmembrane ion permeation pathway unaided. Therefore, CLICs must also undergo an oligomerization process in order to form active ion channels. Our results indicate that oxidative conditions possibly play a key role in promoting the multimerization of mCLIC6. Indeed, while exhibiting oligomerization recalcitrant behavior in solution, suggesting a steric hindrance mechanism preventing surface exposed cysteines from forming intermolecular disulfide bridges (Fig. 7b), TSA experiments demonstrated that exposure to H_2_O_2_ increases the hydrophobicity of mCLIC6 (Fig. 6b). Moreover, the lower *K*_SV_ value obtained from acrylamide-induced quenching of tryptophan fluorescence of mCLIC6 in the presence of H_2_O_2_ (Fig. 6d), indicates reduced solvent accessibility which may originate from oxidation-mediated conformational rearrangements. These observations likely stem from a tertiary structural reorganization, as far-UV CD spectroscopy failed to demonstrate appreciable oxidation-dependent alteration of the secondary structure of mCLIC6 (Supplementary Figure 5).

Cross-linking of mCLIC6 indicate it is capable of undergoing weak oligomerization in the presence of LUVs. Importantly, this oligomerization is visibly augmented in the presence of H_2_O_2_, giving rise to discrete high molecular weight protein species (Fig. 7c). Indeed, fluorescence measurements confirmed that such conditions were necessary to produce significant changes to the local environment of tryptophan residues present in the TRX and α domains (Fig. 7d). Intriguingly, Förster resonance energy transfer was previously used to demonstrate enhanced proximity between W35 of CLIC1 and dansyl-PE containing LUVs under oxidative conditions^33^, reminiscent of mCLIC6 behavior. As these results indicate that both membrane mimetics and oxidative environment are needed to mCLIC6 oligomerization, the question concerning the stoichiometry of an intact CLIC channel still remains. While ion channel formation is a multi-step and highly controlled process of subunit multimerization around a centrally located ion conductive pore^55^, it may involve the incremented formation of lower stoichiometry sub-populations, which gradually assemble into a functional channel^56,57^. Therefore, in light of the previously established oxidation-mediated dimerization of CLIC1^20^, the emergence of higher molecular weight species in solution following exposure to an oxidative environment (Fig. 7b), or the appearance of pronounced protein band corresponding to dimers following cross-linking in the presence of H_2_O_2_ and LUVs (Fig. 7c), suggest subunit dimerization as an obligatory stepping stone towards formation of a membrane-associated CLIC channel.

Hence, we propose a model in which CLICs oligomerization and membrane interaction is a multistep process. First, environmental changes such as an increase in oxidative pressure enhance the frequency of extensive tertiary changes, which include an exposure of the TRX-α domain interface, ‘priming’ CLICs for interaction with the membrane. Next, the ‘primed’ subunits interact with the membrane, forming oligomeric interactions which are maintained and stabilized by the presence of lipids.

In conclusion, we provide here the high-resolution structure of the canonical CLIC domain from mouse CLIC6. Furthermore, using SAXS we demonstrate that mCLIC6 is a highly flexible protein, able to sample multiple distinct conformations in solution. Moreover, perturbation of its inter-domain interface results in a population shift towards elongated conformations. Finally, exposure of mCLIC6 to oxidative conditions results in a significant surface hydrophobicity increase, which likely plays a key role in membrane-induced oligomerization.

## Methods

### Overexpression and purification of mCLIC6

Mouse CLIC6 (Genebank accession code BC075706.1) CLIC domain (residues 363–597; termed mCLIC6) was sub-cloned using 5′ NcoI and 3′ HindIII sites into a pETM11 vector containing an N-terminal hexa-histidine affinity tag followed by a tobacco etch virus protease (TEV) cleavage site. Q383A was introduced using the standard QuickChange approach. Constructs had a GAMG cloning artifact sequence introduced at its amino terminus and were verified by sequencing. mCLIC6 (WT and Q383A) were expressed in T7 Express *E. coli* cells (New England Biolabs) as previously described^58^. Bacterial cultures were grown to mid-log phase and induced with 0.25 mM isopropyl β-D^−1^-thiogalactopyranoside (IPTG) overnight at 16 °C. Cells were harvested and re-suspended in 250 mM NaCl, 50 mM Tris-HCl and 1 mM tris(2-carboxyethyl)phosphine (TCEP), pH 8.0 (buffer A), supplemented with 15 mM imidazole and containing 2.5 μg ml^−1^ DNaseI, 5 mM MgCl_2_, 10 mg lysozyme (Fisher Scientific), 1 mM phenylmethane sulfonyl fluoride and Protease Inhibitor Cocktail Set III (Calbiochem). Cells were lysed with an EmulsiFlex C-5 homogenizer (Avestin) and the lysate was cleared by centrifugation at 32,000 g for 45 minutes at 4 °C. mCLIC6 was then loaded onto a Ni^2+^ affinity resin column (HisTrap HP, GE Healthcare) followed by washing with buffer A containing 27 mM imidazole and eluted using buffer A supplemented with 300 mM imidazole. The hexa-histidine tag was removed by TEV cleavage overnight at 4 °C. Imidazole was removed using a HiPrep 26/10 desalting column (GE Healthcare) equilibrated with buffer A with 15 mM imidazole. The cleaved protein was loaded onto a second Ni^2+^ column with 15 mM imidazole to remove the cleaved hexa-histidine tag and TEV protease. The flow-through was collected, concentrated to 4–5 mL, and loaded onto a HiLoad 16/60 Superdex 75 column (GE Healthcare) equilibrated with gel filtration buffer, containing 150 mM NaCl, 20 mM Na^+^-HEPES, pH 7.5. Finally, pooled fractions were concentrated to ~800 μM using a 10-kD molecular weight cut-off concentrator (Millipore), flash-frozen in liquid N_2_ and stored at −80 °C until use.

### Protein crystallization and data collection

Crystals of mCLIC6 were grown at 19 °C using sitting-drop vapor diffusion by mixing a 2:1 ratio (Mosquito, TTP Labtech) of protein solution at 390 μM and a reservoir solution, containing 23–27% (w/v) PEG 3350, 0–0.2 M LiSO_4_ and 0.1 M Bis-Tris, pH 5.5–6.5, supplemented with 0.1 M glycine or 3% (v/v) methanol. This condition produced crystals within a day, which grow to maximum size of about 200 × 100 × 100 μm after 7 days. For diffraction data collection, crystals were immersed in liquid N_2_ after cryoprotection with either 20% glycerol or monoethylene glycol. Data were collected at 100 K on beamline ID23 of the European Synchrotron Radiation Facility (ESRF; Grenoble, France), using a wavelength of 0.967 Å. Integration, scaling and merging of the diffraction data were done with the XDS program^59^. The crystals belonged to space group P 2_1_ (a = 42.62 Å, b = 68.99 Å and c = 80.98 Å with α = γ = 90° and β = 95.14°) and C 1 2 1 (a = 120.38 Å, b = 51.64 Å and c = 101.10 Å with α = γ = 90° and β = 119.62). As both datasets were anisotropic, an ellipsoidal truncation and anisotropic scaling were performed^60^ using resolution cutoffs of a* = 1.9 Å, b* = 2.0 Å, c* = 1.8 Å (p 2_1_) and a* = 2.2 Å, b* = 1.9 Å, c* = 2.0 Å (C 1 2 1).

### Structure determination

The structure was solved by molecular replacement using the programs PHASER^61^ and PHENIX^62^ with the structure of the soluble form of CLIC4 (PDB accession 2AHE) as the search model. Data collection and refinement statistics are summarized in Table 1. Each asymmetric unit contained two protein subunits. Electron density was visible for most of the protein chains except for a few flexible residues in the β2-β3 loop and protein terminals in some of the chains. Iterative model building and refinement were carried out in PHENIX with manual adjustments using COOT^63^. The final models were refined to a resolution of 1.8 Å (P 2_1_) and 1.9 Å (C 1 2 1), with *R*_work_/*R*_free_ of 0.178/0.217 and 0.204/0.242, respectively. The majority (98%) of the residues are in the most favored region in the Ramachandran plot. All structural illustrations were prepared with UCSF Chimera (https://www.cgl.ucsf.edu/chimera).

### SAXS Data Collection and Analysis

SAXS data were measured at beamline BM29 of the European Synchrotron Radiation Facility (ESRF), Grenoble, France. Data were collected at 5 °C with X-ray beam at wavelength λ = 1.0 Å, and the distance from the sample to detector (PILATUS 1 M, Dectris Ltd) was 2.867 meters, covering a scattering vector range (q = 4 πsinθ/λ) from 0.0025 to 0.5 Å^−1^. 10 frames of two-dimensional images were recorded for each buffer or sample, with an exposure time of 1 s per frame. The 2D images were reduced to one-dimensional scattering profiles and the scattering of the buffer was subtracted from the sample profile using the software on site. Samples were analyzed in gel filtration buffer. To account for possible inter-particle effects, each sample was measured at three concentrations (Table 2). The lowest concentration curve was merged with a higher concentration curve at q ~ 0.2 Å^−1^ to prevent distortion of the low-angle data while preserving high signal-to-noise ratio at the higher angles, which are far less sensitive to interparticle effects^64^. The experimental radius of gyration (Rg) was calculated from data at low q values in the range of *qR*_*g*_ < 1.3, according to the Guinier approximation: lnI(q) ≈ ln(*I*(0)) − *Rg*^2^*q*^2^/3 using PRIMUS^65^. The D_max_ values and the Porod volume were derived from the paired-distance distribution function (PDDF or *P(r)*) calculated using GNOM^66^. *Ab initio* shape reconstructions were performed using the ATSAS software suite, using scattering data between 0.02 and 0.30 Å^−1^ of the q range^66^. The solution scattering of the crystal structure was calculated using CRYSOL^27^. Missing loops from the crystal structure were generated using MODELLER^67^ and the solution scattering of the model was calculated using CRYSOL^27^.

### Ensemble optimization method (EOM) analysis

The software RANCH^28^ was used to generate a pool of 10,000 stereochemically feasible structures with a random linker between the TRX and α-domains. This pool was used as input for GAJOE^28^ that selects an ensemble with the best fit to the experimental data using a genetic algorithm: 50 ensembles of 20 orientations each were “crossed” and “mutated” for 1,000 generations and the process was repeated 50 times.

### Analytical size-exclusion chromatography

Experiments were performed using an analytical gel filtration column (Superdex 200 increase 10/300 GL column, GE Healthcare). Protein samples were thawed, centrifuged at 21,000 g for 10 minutes, and loaded (200 ug) onto a column pre-equilibrated with gel filtration buffer, using an ultra-performance liquid chromatograph system (Shimadzu Corporation).

### Differential scanning fluorimetry

Thermal shift assay was performed in a Real-Time PCR system (StepOne Plus, Life Technologies), with the fluorescent dye SYPRO Orange (Invitrogen, Life Technologies), using the ROX filter set in clear 96 well plates. The temperature was increased using a continuous ramp at a rate of 1 °C/minute from 25 °C to 95 °C. Assays were performed in a 20 µl reaction final volume, containing 3.7 μM protein, 5X SYPRO Orange,150 mM NaCl, 20 mM Na-HEPES (pH 7.5) buffer and different H_2_O_2_ concentrations, in the presence or absence of 5 mM DTT. Melting temperatures were extracted by Boltzmann sigmoidal fitting of the ascending phase of the curves. *T*m dependence of the H_2_O_2_ concentration was fitted using an exponential one phase decay equation (GraphPad Prism).

### Circular Dichroism

37 μM of mCLIC6 in CD buffer, containing 37.5 mM NaCl, 5 mM Na-HEPES (pH 7.5), in the presence or absence of 2 mM H_2_O_2_, were incubated at 4 °C for 10 minutes prior to analysis. Far-UV CD spectra were recorded using an Applied Photophysics Circular Dichroism Spectrometer at room temperature. CD data were collected over the wavelength range of 190–260 nm. Final spectra were the average of three scans, and were baseline-corrected and smoothed. The secondary structure element content was estimated using the CONTIN algorithm^68^ in the DichroWeb online server (dichroweb.cryst.bbk.ac.uk).

### Trp Fluorescence Quenching by Acrylamide

20 µM mCLIC6 in buffer, containing 150 mM NaCl, 20 mM Na-HEPES (pH 7.5), in the presence or absence of 2 mM H_2_O_2_, were incubated at 4 °C for 20 minutes prior to analysis. Acrylamide was then added from an aqueous 5 M stock solution, resulting in concentration gradient ranging between 0 and 250 mM. Fluorescence was measured after 30 minutes incubation in RT. The excitation wavelength was 290 nm, and the observed fluorescence emission was recorded at 345 nm (n = 3). Linear regression was performed using the Stern–Volmer equation for a dynamic process:

1 $$\frac{{F}_{0}}{F}=1+{{K}}_{{SV}}[{Q}]$$

where *F*_0_ is the fluorescence intensity in the absence of acrylamide, *F* is the fluorescence emission as recorded at each concentration of acrylamide, and *Q* is the quenching agent concentration. *K*_SV_ is the Stern–Volmer quenching constant, which is a measure of the accessibility of the tryptophan to the quenching agent.

### Preparation of sucrose-loaded liposomes

10 mg of chloroform dissolved *E. coli* Total lipid Extract (Avanti Polar Lipids), were dried under high vaccum for 45 minutes, followed by rehydration with 1.1 mL of buffer SKH, containing 176 mM sucrose, 100 mM KCl and 20 mM Na-HEPES (pH 7.5). An ultrasonic bath was used to produce multilamellar vesicles, which were further extruded using a 0.2 µM membrane to form homogeneous, sucrose loaded, LUVs. The resulting solution was diluted 5-fold in sucrose omitted buffer SKH (buffer KH). The suspension was next centrifuged at 137,000 g for 1 hour at 4 °C (SW55Ti rotor, Beckman OPTIMA-80 K). The pellet was resuspended in 100 µL of buffer KH by vigorous vortexing at a final lipid concentration of 100 mg/ml.

### Cross-linking oligomerization analysis

Cross-linking experiments of 8 µM mCLIC6, in the presence of 0–400 µg LUVs, were performed using 1 mM disuccinimidyl suberate (DSS) at 4 °C for 2 hours in binding buffer containing 150 mM NaCl, 20 mM Na-HEPES (pH 7.5), in the presence or absence of 2 mM H_2_O_2_. Reactions were stopped by the addition of sodium dodecyl sulfate- and β-mercaptoethanol-containing sample buffer, followed by 10 min incubation at room temperature. Cross-linked products were analyzed by SDS-PAGE using 4–20% Tris-Glycine gels (Bio-Rad).

### Kinetic tryptophan fluorescence measurements

10 mg of chloroform dissolved *E. coli* Total lipid Extract (Avanti Polar Lipids), were dried under high vaccum for 45 minutes, followed by rehydration with 1.1 mL of assay buffer (150 mM NaCl, 1 mM EDTA, and 50 mM Na3PO4 (pH 6.0)). An ultrasonic bath was used to produce multilamellar vesicles, which were further extruded using a 0.2 µM membrane to generate LUVs. Experiments were carried out in a 100 µl reaction final volume, containing 20 µM mCLIC6 in the assay buffer, in presence or absence of 2.5 mM LUVs or 2 mM H_2_O_2_ (n = 5–7). Fluorescence kinetics were performed using a SpectraMax M5 plate reader, where the samples were excited at 290 nm, with emission recorded at 340 nm, and presented as *F*/*F*_o_.

### Conservation Analysis

As CLICs’ sequences of are highly conserved, the crystallized mCLIC6 sequence was used as a BLAST search query (PSI-BLAST threshold E < 0.005), limited by a model organism test set (74 orthologues), as established by the Ensembl server^69^. The resulting 454 hits were used to generate a multiple sequence alignment using Clustalw^70^, followed by Consurf ^54^ analysis, which outputs a conservation score for each residue.

### Accession numbers

Atomic coordinates and structure factors have been deposited in the Protein Data Bank with accession numbers 6ERY and 6ERZ.

## Electronic supplementary material

Supplementary information
