# Supplementary material for: Inherent flexibility of CLIC6 revealed by crystallographic and solution studies
Source: Sci Rep. 2018 May 2;8:6882. doi: 10.1038/s41598-018-25231-z (PMC5931990; doi:10.1038/s41598-018-25231-z)

Supplementary information

Inherent flexibly of CLIC6 revealed by crystallographic and solution studies

Alisa Ferofontov, Roi Strulovich, Milit Marom, Moshe Giladi, and Yoni Haitin

Supplementary Figure legends

Supplementary Figure 1. Asymmetric unit of the C1 2 1 from and crystal packing comparison. (a) Cartoon representation of the asymmetric unit from the C1 2 1 crystal form showing chain A and B in cyan and green, respectively. **(b and c)** Crystal packing of mCLIC6, crystallized in $P2_1$ and C1 2 1 space groups, respectively. A single asymmetric unit is colored as in (**a**).

Supplementary Figure 2. Spatial comparison of mCLIC6 and selected available CLIC structures. (a) Superposition of mCLIC6 with CLIC1 (PDB 1K0N), CLIC2 (PDB 2PER), CLIC3 (PDB 3KJY), and CLIC4 (PDB 2AHE). **(b)** All atom superposition r.m.s.d. values of the corresponding structures.

Supplementary Figure 3. Sequence similarity of mCLIC6 to human CLICs. Sequence alignment of mCLIC6 and human members of the CLIC family. Secondary structure of mCLIC6 is indicated above the sequence. Loops and non-helical secondary structure are marked as solid gray lines. Identical and similar residues are shown in green and grey, respectively.

Supplementary Figure 4. TSA analysis of mCLIC6 in the presence of DTT. Normalized fluorescence-temperature relation of mCLIC6 in the presence of increasing concentrations of H_2O_2 and 5 mM DTT. Boltzmann fits used to calculate *T*m are colored according to H_2O_2 concentrations.

Supplementary Figure 5. Circular dichroism analysis of mCLIC6. Far-UV CD spectra recorded in the absence or presence of 2 mM H_2O_2.

Supplementary Figure 6. mCLIC6 cancer-associated positions conservation. Cancer associated positions in mCLIC6 colored according to Consurf scores.

Supplementary Figure 7. Uncropped protein gel image. Uncropped SDS-PAGE Tris-Glycine (4-20%) protein gel image, used to produce Figure 6f.

Ferofontov et al. Supplementary Fig. S1

a

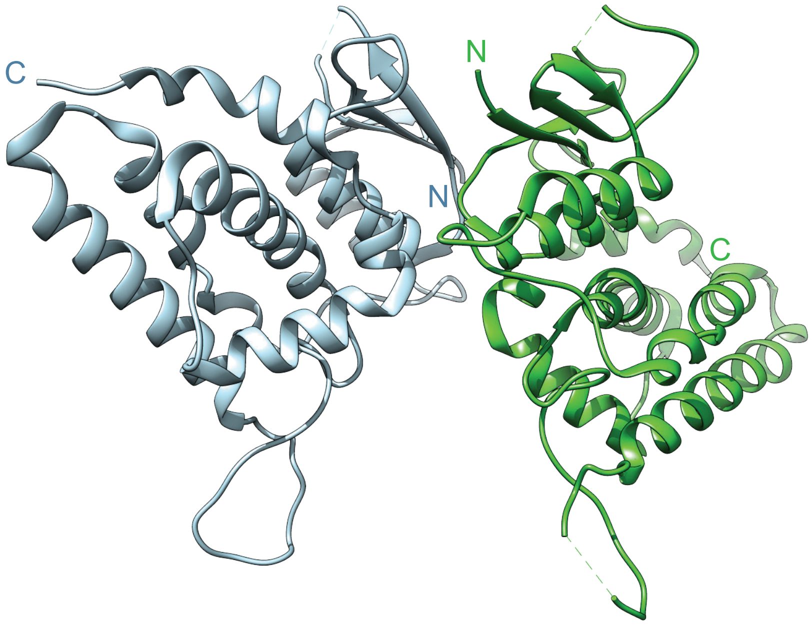

b

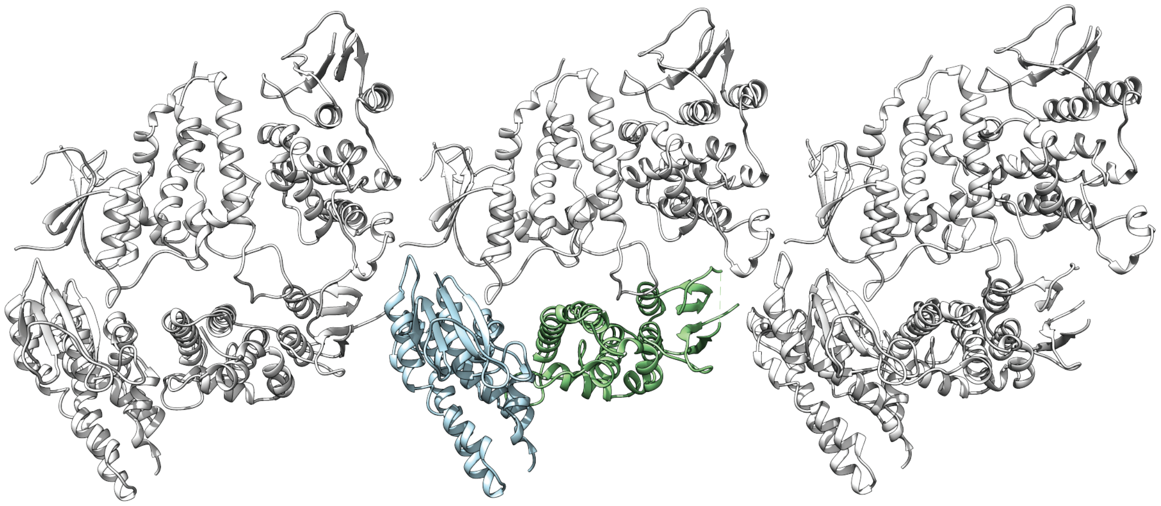

c

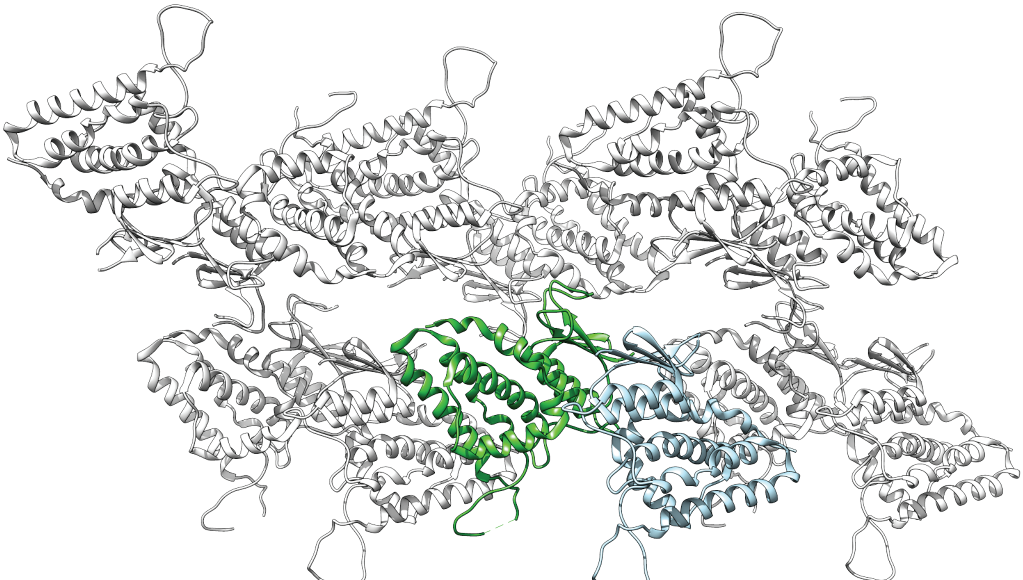

Ferofontov et al. Supplementary Fig. S2

a

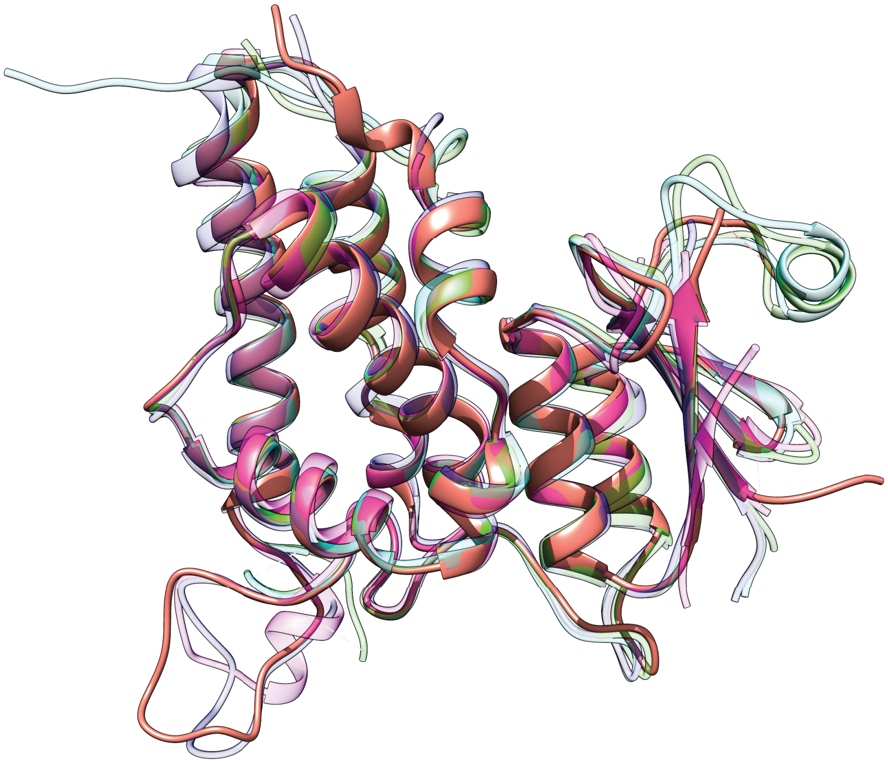

CLIC6 CLIC1 CLIC2 CLIC3 CLIC4

b

	CLIC6	CLIC1	CLIC2	CLIC3
CLIC6				
CLIC1	1.93			
CLIC2	1.83	1.15		
CLIC3	1.46	1.29	2.13	
CLIC4	1.74	0.73	0.88	1.41

Units = Å

Ferofontov et al. Supplementary Fig. S3

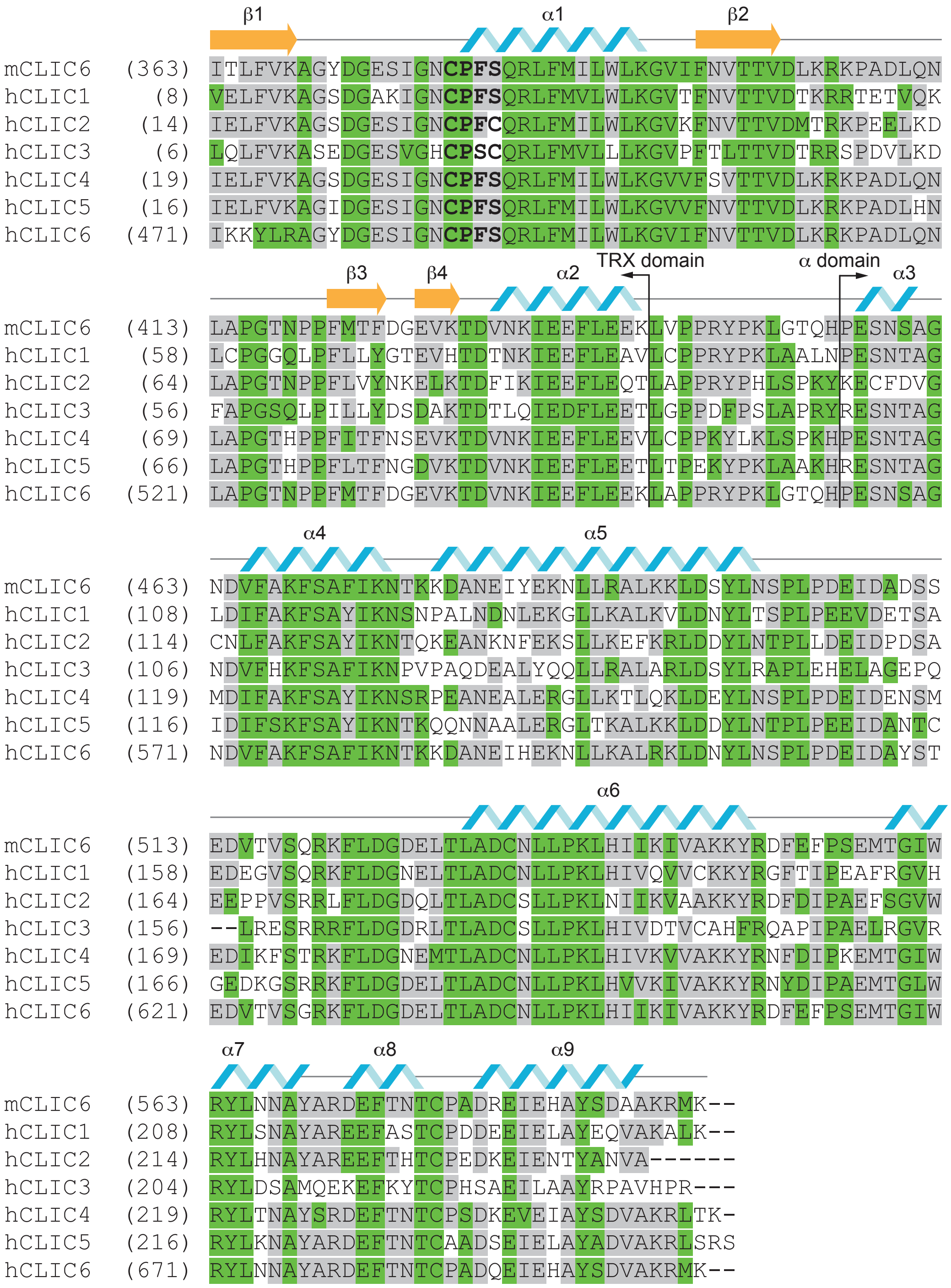

Ferofontov et al. Supplementary Fig. S4

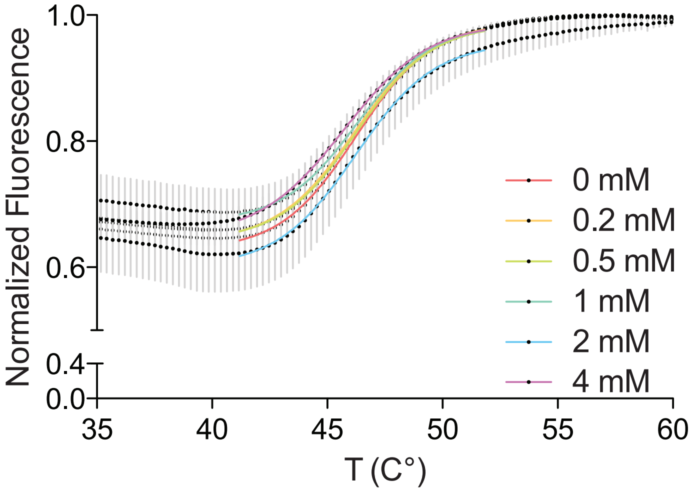

Ferofontov et al. Supplementary Fig. S5

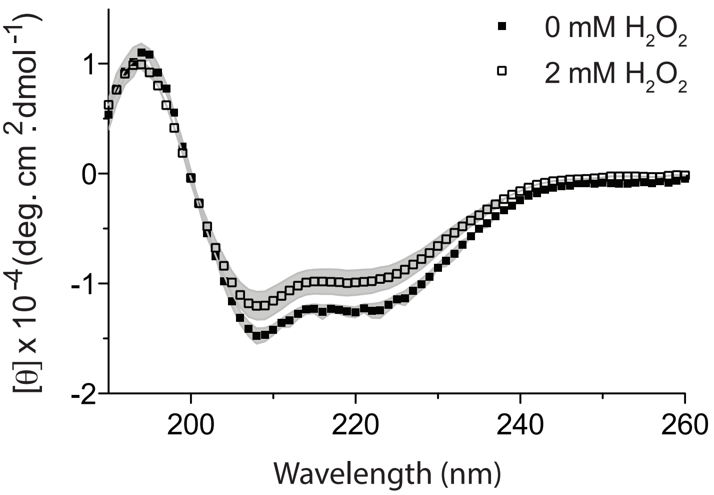

Ferofontov et al. Supplementary Fig. S6

Position hCLIC6	Position mCLIC6	Human isoform	Mutation	Consurf score
463	373	A	G>S	-0.218
467	377	A	G>E	-0.562
472	382	A	S>Y	-0.645
474	384	A,B	R>H	-0.949
475	385	A,B	L>P	-0.404
479	389	A,B	L>F	-0.538
492	402	A,B	D>N	-0.979
498	408	A	A>T	1.134
499	409	A	D>V	0.065
504	414	A,B	A>T	-0.652
507	417	A,B	T>K	-0.121
516	426	A,B	G>D	1.59
524	434	A,B	K>T	-0.82
526	436	A	E>K	-0.562
539	449	A	P>A	-0.042
545	455	A	H>R	0.125
546	456	A,B	P>S	0.636
547	457	A	E>K	-0.504
550	460	A	S>C	-0.04
551	461	A,B	A>T	-0.454
555	465	A	V>M	-0.146
559	469	A,B	F>L	-0.883
561	471	A,B	A>V	-0.82
566	476	A	T>M	-0.113
572	482	A	E>K	2.75
575	485	A	E>K	0.241
576	486	A	K>N	0.351
586	496	A,B	D>Y	-0.679
591	501	A	S>C	0.916
596	506	A	E>K	-0.895
601	511	A	S>T	-0.229
619	529	A,B	T>M	-0.942
630	540	A,B	H>Y	-0.397
641	551	A	D>E	0.823
645	555	A,B	P>S	-1.137
653	563	A	R>G	-0.431
658	568	A,**B**	A>S **A>V**	-1.023
661	571	**A,B**	**R>G R>K**	0.627
662	572	A,B	D>N	0.092
663	573	A,B	E>A	-0.837
664	574	A,B	F>L	-1.096
673	583	A,B	E>Q	-1.19
685	595	A,B	M>I	-0.517

Key	
Variable	1
	2
	3
	4
	5
	6
	7
	8
Conserved	9

Ferofontov et al. Supplementary Fig. S7

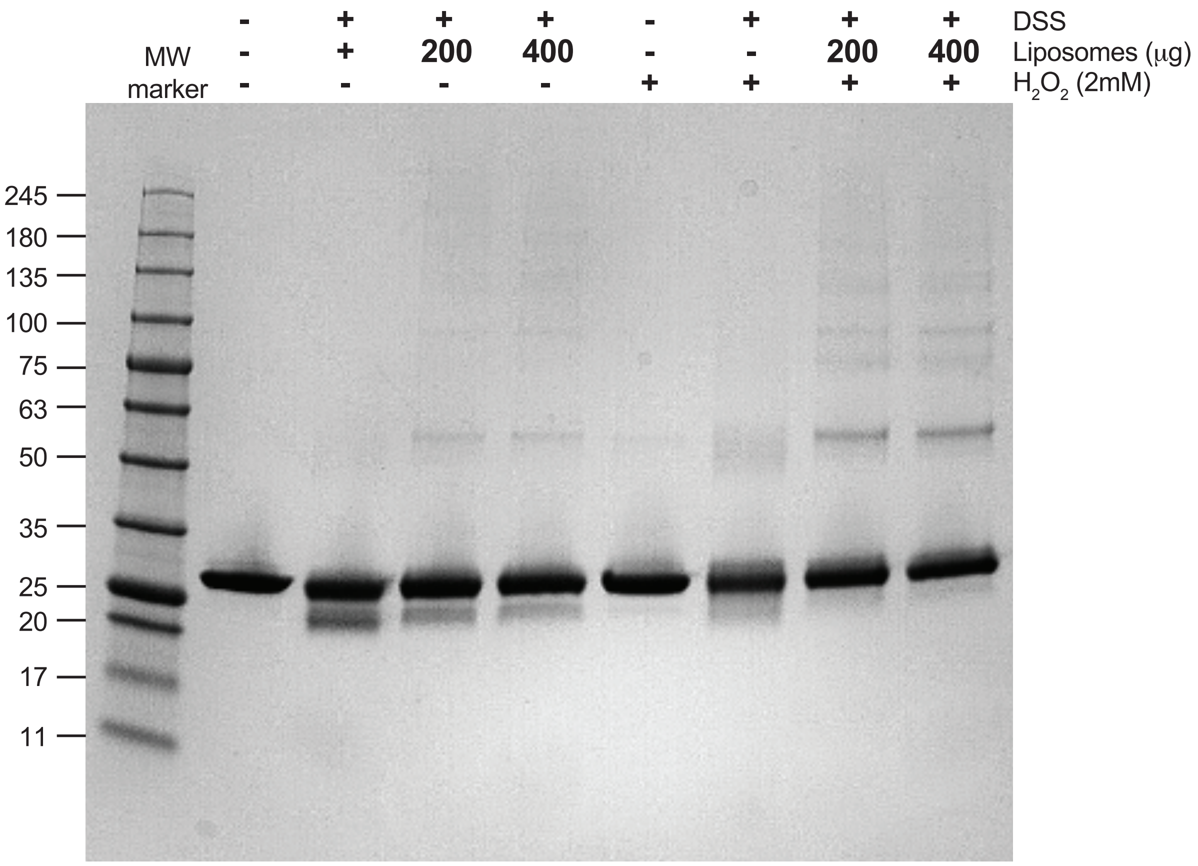

Supplement: Supplementary file 1 — Supplementary information [file 41598_2018_25231_MOESM1_ESM.pdf]
